# Demographics, referral patterns and management of patients accessing the Welsh Eye Care Service

**DOI:** 10.1186/s40662-016-0045-7

**Published:** 2016-05-18

**Authors:** Colm McAlinden, Helen Corson, Nik Sheen, Peter Garwood

**Affiliations:** Public Health Wales, Floor 11, The Oldway Centre, 36 Orchard Street, Swansea, SA1 5AQ UK; Public Health Wales, Cwmbran House, Mamhilad Park Estate, Pontypool, Torfaen, NP4 0XS UK; School of Optometry and Vision Sciences, Cardiff University, Maindy Road, Cardiff, CF24 4 LU UK

**Keywords:** Wales eye care services, Primary eyecare assessment & referral service, Welsh eye care initiative, Welsh eye health examination

## Abstract

**Background:**

The Primary Eyecare Acute Referral Service (PEARS) and the Wales Eye Health Examination (WEHE) operate as enhanced optometry services for patients residing in Wales, enabling the examination of a patient presenting with an acute eye problem (PEARS) or the examination of patients at higher risk of eye disease (WEHE). The purpose of the study is to assess the demographics of patients accessing these services, referral patterns and clinical management in one Health Board in Wales (Aneurin Bevan University Health Board).

**Methods:**

Information from 2302 patients accessing the services was prospectively collected. The following information was obtained: type of examination (PEARS or WEHE), patient age, gender, self-referral or general practitioner (GP) referral and clinical management (no further action, monitor by optometrist or ophthalmic medical practitioner [OMP], refer to the Hospital Eye Service [HES], or refer to GP).

**Results:**

There were 1791 (77.8 %) PEARS examinations and 511 (22.2 %) WEHE. There were 1379 (59.9 %) females with a mean age of 58.61 (±19.75) and 923 (40.1 %) males with a mean age of 56.11 (±20.42). The majority of patients were self-referrals compared to GP-referrals (1793 [77.9 %] versus 509 [22.1 %] respectively). Sub-analysis indicated similar numbers of self-referrals compared to GP-referrals for the WEHE only (297 [58.1 %] versus 214 [41.9 %] respectively) but greater numbers of self-referrals for the PEARS examinations only (1496 [83.5 %] versus 295 [16.5 %] respectively). For management, 75 % of patients were monitored by their optometrist or OMP, 17 % required referral to the HES and 8 % required referral to their GP.

**Conclusions:**

Higher numbers of females accessed both PEARS and WEHE services and the majority of patients self-referred. These findings have important implications for public health campaigns both for targeting specific groups (e.g. male patients) and increasing awareness among GPs.

## Background

The Primary Eyecare Acute Referral Service (PEARS) and the Wales Eye Health Examination (WEHE) were enhanced optometry based services that had operated for 10 years for patients residing in Wales [1]. Patients generally either self-refer or are referred by their General Practitioner (GP) to accredited practitioners in community optometry practices. Patients requiring a PEARS examination are able to be seen within 24 h and undergo appropriate investigations at the discretion of the practitioner while patients attending for a WEHE undergo standard predetermined ophthalmic investigations [1]. These services were funded by the Welsh Government at no cost to the patient, enabling the examination of a patient presenting with an acute eye problem (PEARS) or the examination of patients at increased risk of eye disease (WEHE). The reimbursement was £60 for a PEARS and £40 for a WEHE. In April 2013, these services were amalgamated into the new Eye Health Examination Wales (EHEW) Service with the core aims of the service kept the same as the previous PEARS and WEHE. The services are part of the wider Welsh Eye Care Service (WECS), which also includes the Low Vision Service Wales and Diabetic Retinopathy Service Wales [2–6]. All the WECS services continue to be updated and further information is available on the website www.eyecare.wales.nhs.uk [7]. In brief, the PEARS and WEHE service core values have been retained within a new Eye Health Examination Wales (EHEW) service launched in 2013. The EHEW service contains a structured framework that operates on a 3-tiered banded system. The Band 1 part of the EHEW service retains all the previous categories of the PEARS and WEHE service in an amalgamated format. The additional Bands 2 and 3 enable community optometrists to further inform or prevent onward referral to the hospital eye service, or carry out a follow up assessment of a patient, respectively.

Optometrists and ophthalmic medical practitioners (OMPs) are required to undergo accreditation, which involves successful completion of distance learning lectures with multiple choice questions, and practical Objective Structured Clinical Examinations (OSCEs).

The purpose of the present study is to assess the demographics of a sample of patients accessing this service, referral patterns to the service and to assess the outcomes of the ophthalmic examinations in terms of management.

## Methods

Following a PEARS examination or WEHE, optometry practices submit a claim form to the National Health Service (NHS) Wales Shared Services Partnership in order to receive payment. The information on the claim forms for 2302 consecutive submissions was prospectively collected in the month of February 2012 from one health board (Aneurin Bevan University Health Board). The following information was obtained from each submitted form: type of examination (PEARS or WEHE), patient age, patient gender, self-referral or GP-referral and the outcome of the examination (no further action, monitor by optometrist or OMP, refer to the Hospital Eye Service [HES], or refer to GP). Practitioners were also required to inform the patient’s GP of the outcomes of the eye examination and there was a tick box on the claim form to reflect if this has been done. The total numbers of patients accessing each service was determined and the mean age, standard deviation and range calculated. The mean, median, standard deviation, range, interquartile range was calculated for patient ages and t-tests used to assess statistical significance of comparison. A *P*-value of 0.05 was the threshold used to infer statistically significant differences.

## Results

**Fig. 1:**
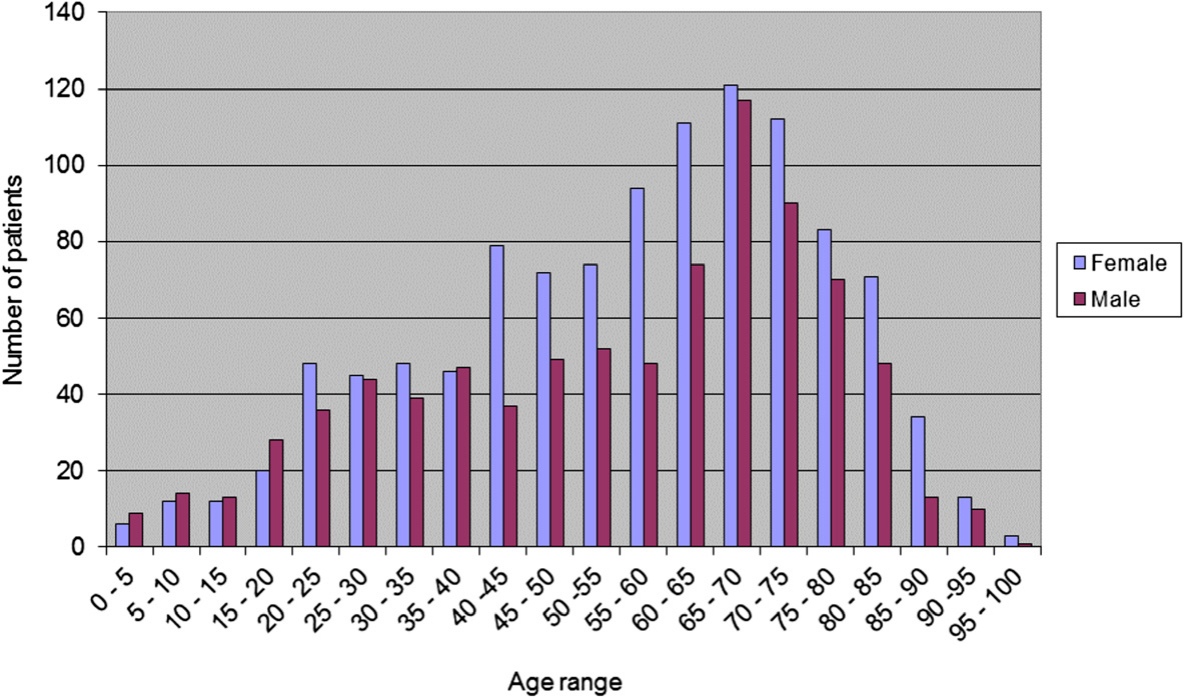
Number of patients by age and gender for the Primary Eyecare Acute Referral Service (PEARS)

**Fig. 2:**
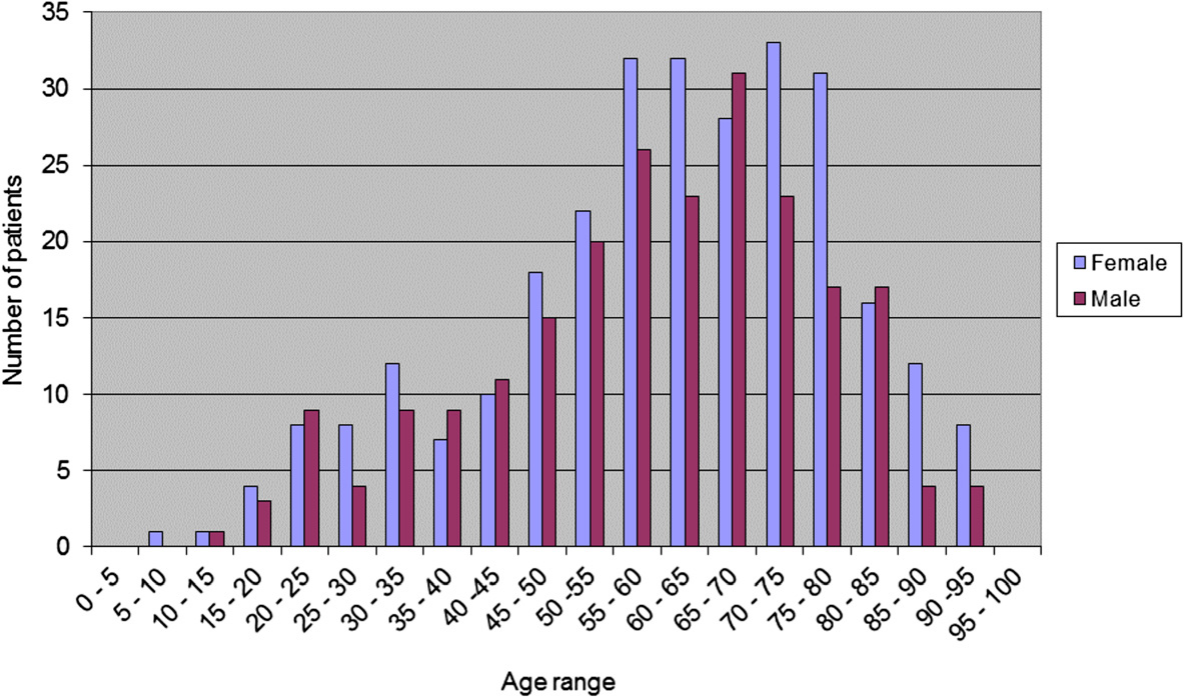
Number of patients by age and gender for the Welsh Eye Health Examination (WEHE)

From the 2302 claim forms reviewed, 1791 (77.8 %) were for PEARS examinations and 511 (22.2 %) were for WEHE. Full demographics are displayed in Table 1. This age difference between females and males was not statistically significant (*P* = 0.08, 95 % CI −0.302 to 5.302). Patients were divided into age vigintiles and are displayed in Fig. 1 (PEARS) and Fig. 2 (WEHE).

**Table 1:** The number, age, standard deviation and range of patients accessing the Primary Eyecare Acute Referral Service (PEARS) and the Wales Eye Health Examination (WEHE) service

PEARS and WEHE					
	Number (n)	Mean (median) age in years	± Standard deviation (years)	Range (years)	Interquartile range (years)
Total	2302	57 (61)	20	2 – 100	29
Female	1379	58.6 (61)	19.8	2 – 97	29
Male	923	56.1 (61)	20.4	3 – 100	31
*P* value	–	0.08	–	–	–
WEHE only					
Total	511	60.5 (63)	17.9	10–95	11.5
Female	283	61.3 (63)	18.2	10–95	24
Male	228	59.6 (62)	17.5	13–93	22.3
*P* value	–	0.28	–	–	–
PEARS only					
Total	1791	56 (60)	20.5	2–100	30
Female	1096	56.7 (60)	20	2–97	29
Male	695	55 (61)	21.2	3–100	34
*P* value	–	0.09	–	–	–

The majority of patients presenting were self-referrals compared to GP-referrals. Sub-analysis indicated similar numbers of self-referrals compared to GP-referrals for the WEHE only while the PEARS only saw significantly greater numbers of self-referrals compared to GP-referrals (Table 2).

**Table 2:** Comparison of self-referral and General Practitioner (GP) referral to community optometry practices

	Total number (%)	WEHE only (%)	PEARS only (%)
Self-referral	1793 (77.9 %)	297 (58.1 %)	1496 (83.5 %)
GP-referral	509 (22.1 %)	214 (41.9 %)	295 (16.5 %)

Table 3 indicates the number of patients managed in the community by optometrists and OMPs compared to the numbers referred to the HES or GP.

**Table 3:** Clinical management outcomes of patients accessing the Primary Eyecare Acute Referral Service (PEARS) and the Wales Eye Health Examination (WEHE) service

	Total number (%)	Total number, WEHE only (%)	Total number, PEARS only (%)
No further action	640 (27.8 %)	265 (51.9 %)	375 (20.9 %)
Monitor by optometrist or OMP	997 (43.3 %)	168 (32.9 %)	829 (46.3 %)
Refer to HES	367 (15.9 %)	52 (10.2 %)	316 (17.6 %)
Refer to GP	168 (7.3 %)	14 (2.7 %)	154 (8.6 %)
Inform GP	1223 (53.1 %)	222 (43.4 %)	1001 (55.9 %)

## Discussion

The Welsh Eye Care Service enables the provision of both urgent eye examinations as well as eye examinations for patients at increased risk of eye disease including cataract, diabetic eye disease, macular degeneration and glaucoma. The main findings from this study were that markedly greater numbers of females than males were found to be utilising the service. Closer inspection of the PEARS and WEHE service individually showed that this marked difference was primarily from the PEARS. There are many possible reasons for these differences. Firstly, one must consider any population difference between females and males in this area. The Aneurin Bevan University Health Board covers the local authority areas of Blaenau Gwent, Caerphilly, Monmouthshire, Newport and Torfaen. The combined population of these areas as reported by the Office of National Statistics in 2011 was 576,754 of which 294,044 were female and 282,710 were male [8]. This equates to 50.98 % female and 49.02 % male, indicating a similar number of females and males in this area. Therefore, the differences in the results found suggest that women are more concerned with acute eye problems than males and/or women are more forthcoming in attending their community optometry practice or GP practice [9, 10]. It may also indicate that males present to the hospital accident and emergency department rather than their community optometry or GP practice and/or that they may be unaware of the PEARS and WEHE service. These findings have important implications for public health campaigns, which should consider greater targeting towards males to increase awareness of the PEARS and WEHE services. Previous studies of patients presenting to an eye accident and emergency department have similar numbers of males and females. A UK based study of 150 patients presenting to eye accident and emergency department had a male:female ratio of 48 %:52 % [11]. A recent large study in the USA involving 11,929,955 visits to the emergency department had a male:female ratio of 54 %:46 % [12].

There was also a noticeable difference in the numbers of patients presenting across the age vigintiles (Figs. 1 and 2) for both the PEARS and WEHE services. Considering the PEARS firstly, the number of patients was low up to the age of 20 for both females and males, doubling after the age of 20 and remaining similar until approximately 40 years of age for both genders. After 40 years of age, there was a significant increase in the number of female patients attending the PEARS examination. In contrast, there was a decrease in the number of male patients presenting in this age group. The number of females remained significantly greater than the number of males between the ages of 40 and 60. At the age of 60, there was a further increase in patients presenting, both for males and females. Further, there was a large increase in the number of males presenting between the age groups 60–65 and 65–75. The number of patients presenting to utilise the service peaks at the 65–70 age group and gradually reduces in the age groups from 70 years onwards. This increase after the age of 60 may be due to an increased awareness of the service following contact with an ophthalmic practice at the age of 60 when a person becomes eligible for a free GOS sight test. Also, with increasing age, there is an increased risk of acute ophthalmic conditions and hence may indicate the higher numbers of service users in these age groups.

The WEHE service maps a slightly different picture. There was a gradual increase in the number of patients with increasing age and generally greater numbers of females in each age vigintile with the exception of 20–25 years, 40–45 years, 65–70 years, and 80–85 years. Age peaks for females were broad, between 55 and 80 years whereas for males, there was a more obvious distinct peak at 65–70 years. Remembering that the WEHE service aims to address those at risk, this trend largely reflects patients who are eligible for the service.

There were greater numbers of patients that self referred to community optometry practices compared to GP referrals. However, looking at the differences between the PEARS and WEHE services, this difference was mostly evident for the PEARS, with a five fold greater number of self-referrals compared to GP referrals. This indicates that the vast majority of patients visited their community optometry practice with an acute eye problem rather than attending their GP practice first. Although an alternative possible explanation for this finding may be that, a patient presented for a sight test with their optometry practice but an acute eye problem was evident and a PEARS examination was claimed by the practice. However, considering the former as the case, this indicates that many patients have become aware of this optometry-based service but perhaps may also reflect less awareness or use of the service by GPs as mentioned above. On a similar point, only 56 % of optometrists and OMPs informed the GP of the outcomes of the PEARS or WEHE in this study. Improving this communication with the GP has a number of advantages; GPs take overall responsibility of the clinical care of their patients and having full information on any interaction with other professionals will improve patient care.

Finally, assessment of the clinical management of the PEARS and WEHE services indicated that 75 % of patients were monitored by their optometrist or OMP with only 17 % requiring referral to the HES. We do not have information on whether any treatments were prescribed or not. Eight percent were referred to their GP. There are a number of advantages of these findings, such as accessibility (socio-organisationally and geographically), continuity of care, [13] shared care services (e.g. glaucoma care) [14] and that many ophthalmic conditions presenting to eye casualty are non-ophthalmic emergencies [11]. Further, the PEARS and WEHE services in the community are less expensive than the HES. If the 75 % of patients managed in the community were referred to the HES, this would be associated with significantly greater and unnecessary costs to the NHS. In addition, the 75 % of patients managed in the community may reflect less serious ophthalmic conditions not requiring secondary care management with only the more complex ophthalmic conditions (17 % of patients) requiring referral for secondary care management. However, what must be considered is that a proportion of these examinations are likely to be for mild self-limiting conditions, which would not be managed even when referred to secondary care. An additional benefit of the service is that patients are able to attend their local optometry practice without the need to travel to a hospital that may be a long distance away, particularly in rural parts of Wales. These findings are similar to the results reported by Sheen and colleagues who assessed the efficacy of the PEARS and WEHE services [1]. They evaluated 6432 consecutive individuals presenting to 274 optometrists over an eight-month period and found that 4243 (66 %) individuals were managed in optometric practice, which was assessed to be an appropriate management step in 99 % of individuals. They also reported that 87 % of the patients travelled less than 5 miles to attend an optometrist to access the service. The hospital notes of 392 individuals were reviewed and 75 % of the referrals were considered appropriate. This equates to an inappropriate management in 25 % of cases but is confounded by cases of posterior vitreous detachment (PVD). Referrals for uncomplicated PVD were classified as inappropriate referrals. However, a number of HES departments in Wales utilise local protocols in which optometrists are required to refer uncomplicated PVD.

There are a number of limitations in the present study. The data collected relates to only one health board area of Wales (Aneurin Bevan University Health Board), so the findings may not be representative of the entire country of Wales. However, the demographics of this health board area are similar to the demographics of the more populated areas of Wales [8]. Another limitation of the study is the basic method of sampling and was conducted over a short duration (month of February), which may not be representative of the year. Despite the basic method of sampling, this has resulted in a large sample size. A similar large sample size may not have been achievable with other sampling methods.

## Conclusion

In conclusion, the main findings of this study are that significantly higher numbers of females are accessing the PEARS and WEHE services than males and there are variations across the age groups. The majority of patients self referred to the service as opposed to GP referrals. These findings have important implications for public health campaigns both for targeting specific groups, increasing awareness among GPs and demonstrating the cost effectiveness and efficacy of this service. Awareness training alerting GPs to the EHEW services is currently in place.
